# Genetic Diversity of *Babesia bovis* MSA-1, MSA-2b and MSA-2c in China

**DOI:** 10.3390/pathogens9060473

**Published:** 2020-06-15

**Authors:** Jinming Wang, Jifei Yang, Shandian Gao, Xiaoxing Wang, Hao Sun, Zhaoyong Lv, Youquan Li, Aihong Liu, Junlong Liu, Jianxun Luo, Guiquan Guan, Hong Yin

**Affiliations:** 1 State Key Laboratory of Veterinary Etiological Biology, Key Laboratory of Veterinary Parasitology of Gansu Province, Lanzhou Veterinary Research Institute, Chinese Academy of Agricultural Sciences, Xujiaping 1, Lanzhou 730046, China; wjm0403@caas.cn (J.W.); yangjifei@caas.cn (J.Y.); gaoshandian@caas.cn (S.G.); wangxiaoxing812@163.com (X.W.); Sunhao9412@163.com (H.S.); lv_zhaoyong@163.com (Z.L.); liyouquan@caas.cn (Y.L.); liuaihong@caas.cn (A.L.); liujunlong@caas.cn (J.L.); luojianxun@caas.cn (J.L.); 2 Jiangsu Co-Innovation Center for the Prevention and Control of Important Animal Infectious Disease and Zoonose, Yangzhou University, Yangzhou 225009, China

**Keywords:** *Babesia bovis*, genetic diversity, merozoite surface antigen, China

## Abstract

The apicomplexan parasite *Babesia bovis* is a tick-borne intracellular hemoprotozoan parasite that is widespread across China. Genetic diversity is an important strategy used by parasites to escape the immune responses of their hosts. In our present study, 575 blood samples, collected from cattle in 10 provinces, were initially screened using a nested PCR (polymerase chain reaction) for detection of *B. bovis* infection. To perform genetic diversity analyses, positive samples were further amplified to obtain sequences of three *B. bovis* merozoite surface antigen genes (MSA-1, MSA-2b, MSA-2c). The results of the nested PCR approach showed that an average of 8.9% (51/575) of cattle were positive for *B. bovis* infection. Phylogenetic analyses of the predicted amino acid sequences revealed that unique antigen variants were formed only by Chinese isolates. Our findings provide vital information for understanding the genetic diversity of *B. bovis* in China.

## 1. Introduction

The phylum Apicomplexa consists of several highly pathogenic tick-borne parasites of domestic animals across the world, such as *Babesia*, the causative agent of bovine babesiosis. This disease is widespread in tropical and subtropical regions of the world, caused by several distinct *Babesia* species (*B. bovis*, *B. bigemina*, *B. ovata*, *B. major*, *B. divergens*, and *B. orentialis*) [1,2]. Except for *B. divergens*, the afore mentioned *Babesia* spp. has been documented to be a major cause of bovine babesiosis in China [3]. In general, *B. bovis* is notorious for being highly pathogenic, causing significant economic loss to the livestock industry, and has been frequently reported in different areas [3,4,5,6]. Although tick control strategies and treatments for babesiosis are available, this disease has always been a dominant cause of loss to the cattle industry. Outbreaks of bovine babesiosis have been previously described in cattle populations that had been immunized with live attenuated vaccine [2]. The genetic diversity of the major antigenic components of the parasites may be the main reason for vaccination inefficiency [7].

The *B. bovis* merozoite surface antigen genes *msa*-1, *msa*-2b, and *msa*-2c genes have been used as genetic markers to investigate the diversity of *B. bovis* strains in several countries [8,9,10]. The proteins encoded by *msa*-1, *msa*-2b, and *msa*-2c genes are members of the variable merozoite surface antigen family, and play important roles during the invasion of red blood cells by *B. bovis* merozoites [2,11,12,13]. It has been confirmed that sera against recombinant forms of MSA-1, MSA-2b and MSA-2c were able to inhibit merozoite invasion, which indicated that MSAs are potential candidates for exploring subunit vaccines [14,15,16]. However, the genetic diversity of MSAs generate antigenic variation among strains, which leads to weak immunological reactivity among different geographic isolates and is thought to be an important reason for outbreaks in immunized cattle populations [7,17]. Thus, full evaluation of MSA polymorphism may provide vital data for developing an effective immunization strategy against *B. bovis* infection.

Phylogenetic analyses of *B. bovis* MSAs have been performed in several countries, including Mongolia, Vietnam, the Philippines, Thailand, Israel, Vietnam, Sri Lanka, Mexico, Argentina and Brazil [7,12,18,19,20,21,22,23,24,25,26]. In China, molecular epidemiological investigations of *B. bovis* infection have been performed in several provinces [4,5]. However, there is little information on the phylogenetic relationships among *B. bovis* MSAs. In the present study, we therefore screened 575 blood samples randomly collected from cattle in 10 provinces to obtain *B. bovis* positive blood DNA samples, in order to analyze the diversity of MSA sequences in these samples.

## 2. Materials and Methods

### 2.1. Sample Collection and DNA Extraction

**Figure 1:**
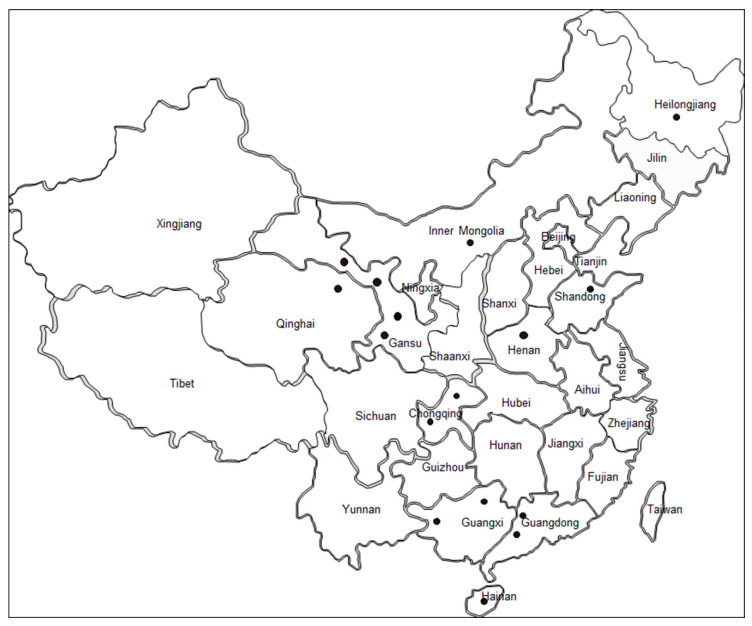
Sample collection sites. Black circles indicate the locations where samples were collected.

Between August 2008 and July 2016, blood samples were randomly collected from cattle (*n* = 575) in 10 provinces of China (Table 1, Figure 1) where bovine babesiosis was previously reported [3,4,5]. These samples were extracted into Ethylene Diamine Tetraacetic Acid (EDTA)-coated vacutainer tubes, transferred to the laboratory in iceboxes and stored at −20 °C before DNA extraction. Subsequently, 200 μL of each blood sample was used for genomic DNA extraction (Qiagen DNA Blood Mini-Kit, Hilden, Germany). The DNA concentration was determined using a NanoDrop 2000 spectrophotometer (NanoDrop Technologies, Wilmington, DE, USA).

### 2.2. Screening Positive Samples for B. bovis Infection

Nested PCR (nPCR) targeting the spherical body protein-2 (SBP-2) was applied to detect cattle infected with *B. bovis,* as previously described (Table 2) [27]. Briefly, in the first round of the PCR, PCR was performed in a total volume of 20 μL, consisting of 2.5 μL of 10 × PCR buffer (Mg^2+^ plus), 2.0 μL of deoxy-ribonucleoside triphosphate (2.5 mM each), 1.25 U of *Taq* DNA polymerase (TaKaRa, Dalian, Liaoning, China), 2.0 μL of template DNA, 1.0 μL of each primers (10 μM) and 16.25 μL of double-distilled water. DNA of *B. bovis* merozoites and double-distilled water were used as a positive control and a negative control, respectively. At the beginning step, PCR mixtures were heated up to 95 °C for 3 min, afterwards a total of 35 cycles of denaturation and an extension were conducted at 95 °C for 30 s, 53 °C for 30 s and at 72 °C for 90 s, respectively. Finally, a final extension reaction was maintained at 72 °C for 5 min. In the second round of PCR, the components of the PCR mixture and reaction parameters were the same as those of the first round PCR, except the template was replaced with first round PCR amplification products and the annealing temperature was maintained at 55 °C. The PCR amplicons were analyzed on a 1.5% agarose gel-containing gold view dye (SolarBio, Beijing, China) in Tris-acetate-EDTA (TAE) buffer at 120 V for 30 min and visualized under ultraviolet light.

### 2.3. MSA-1, MSA-2b and MSA-2b Gene Amplification and Sequencing

Three sets of primer pairs were applied for amplification of the *B. bovis msa-1*, *msa-2b*, and *msa-2c* genes. Procedures of PCR were the same as previously reported [23]. Before amplicon was cloned into a pGEM-T Easy vector (Promega, Madison, WI, USA), PCR amplification amplicons of positive samples infected with *B. bovis* were purified with Gel DNA Recovery Kit (Zymo, Irvine, CA, USA). Subsequently, recombinant vectors were transformed into *Escherichia coli* DH5a competent cells. Three recombinant clones bearing the target gene of each sample were selected and sequenced using BigDye Terminator Mix (Genscript, Nanjing, Jiangsu, China).

### 2.4. Phylogenetic Analyses

The *msa-1*, *msa-2b* and *msa-2c* gene sequences obtained in this study, together with those previously submitted to the GenBank, were translated into amino acid and aligned using the ClustalW program in MEGA 6.0. These sequences were trimmed into common parts and the final sizes of MSA-1, MSA-2b and MSA-2c alignments were 297, 158 and 237 amino acids, respectively. Three phylogenetic trees were generated using a maximum likelihood phylogeny, based on a General Time Reversible and JTT matrix substitution models, using MEGA 6.0 [28]. Representative sequences of distinct clades were submitted to the GenBank database.

## 3. Results

### 3.1. Diagnosis of B. bovis Infection in Cattle

The investigation of 575 field blood samples, obtained from cattle in 10 provinces across China, using the nested PCR approach, illustrated an average of 8.9% (51/575) of cattle infected with *B. bovis* (Table 1, Figure 1). *Babesia bovis* infection was present in all 10 provinces investigated. In particular, Guangdong, Inner Mongolia and Hainan provinces were determined as relatively high risk areas of *B. bovis* infection, with a positive rate of 15.3%, 25%, and 10.2%, respectively. In other provinces, less than 10% of the cattle population was determined to have *B. bovis* infection. The lowest percentage of infection was observed in Guangxi Province.

### 3.2. DNA Sequencing of MSA-1, MSA-2b, and MSA-2c Genes

Only 21 out of 51 positive samples, including 13 samples for MSA-1, 7 for MSA-2b, and 8 for MSA-2c, were successfully amplified for subsequent analysis (Table 2). Sequence analysis revealed that all samples were successfully cloned and sequenced. The length of PCR amplicons of the partial *msa-1*, *msa-2b* and *msa-2c* genes ranged from 924 to 960 base pairs (bp), 669 to 732 bp, and 711 to 717 bp, respectively. Three clones were sequenced for each sample; however, the sequences of two amplicons were not identical (Table 2). Representative sequences for *msa-1*, *msa-2b* and *msa-2c* were submitted to the GenBank database under accession numbers MT113038–MT113065. Predicted amino acid sequences of MSAs were aligned using a CLUSTAL 0 (1.2.4) (http://clustal.org/omega/) (Supplementary File S1–S3).

### 3.3. Phylogenetic Analyses of MSA-1, MSA-2b and MSA-2c Genes

**Figure 2:**
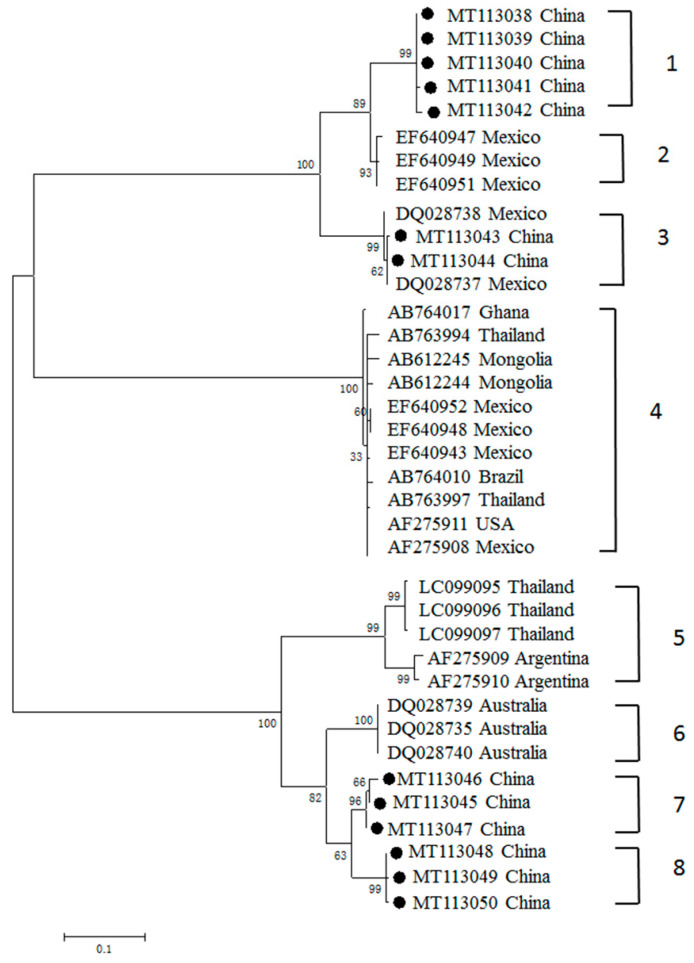
Phylogenetic tree of the predicted amino acid sequences of *B. bovis msa-1* gene obtained in this study (labeled with black circles), together with other sequences previously deposited in the GenBank.

Three phylogenetic trees were generated using 13, 7 and 8 sequences obtained in this study, along with 24, 27 and 37 sequences of *B. bovis* MSA-1, MSA-2b and MSA-2c genes previously submitted to the GenBank database, respectively. Phylogenetic analysis showed that the *B. bovis msa-1* genes generated in our study were divided into four clusters, among which three were completely formed by Chinese sequences generated in this study (Figure 2). The percentages of nucleotide identity among the *MSA-1* sequences varied from 62.6% to 99.8%, while the percentages of amino acid identities varied from 43.8% to 100% (Supplementary Table S1). The percentages of nucleotide identity within clusters ranged from 98.9% to 99.8%; the maximum and the minimum were observed in cluster 1 and cluster 8, respectively.

**Figure 3:**
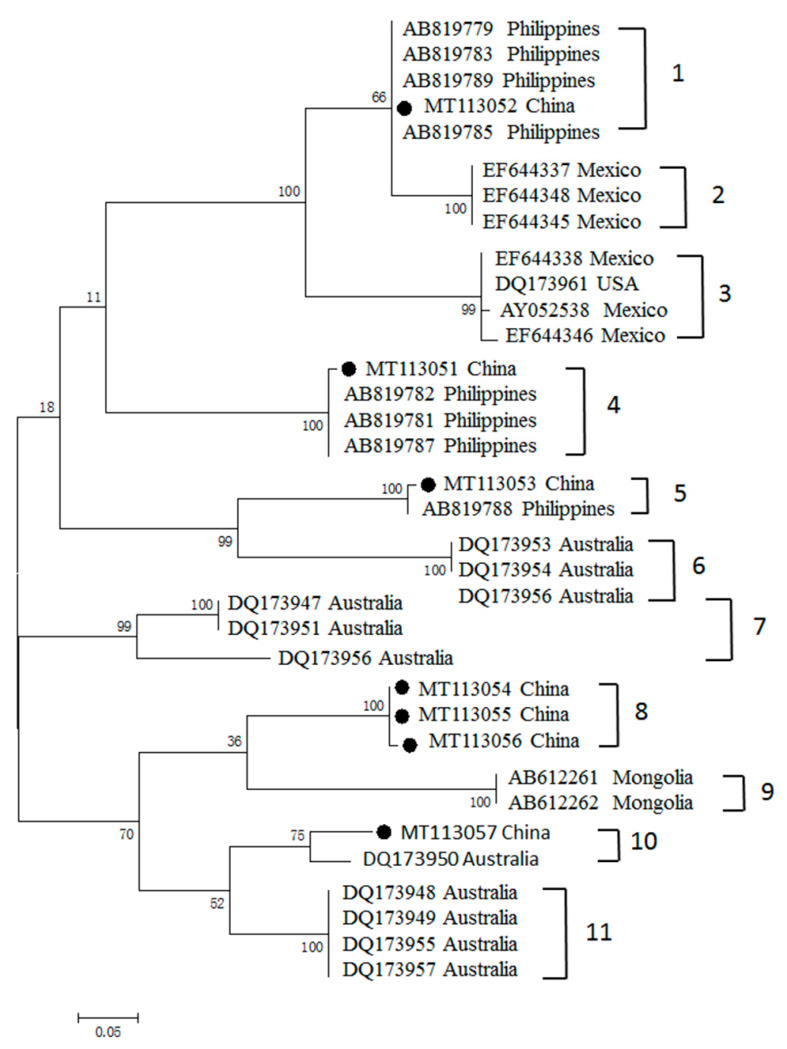
Phylogenetic tree of the predicted amino acid sequences of *B. bovis* MSA-2b and sequences previously deposited in the GenBank. The MSA-2b sequences obtained in this study are labeled with black circles.

The analysis of phylogenetic relationships illustrated that the MSA-2b sequences obtained in this study were well-separated into five clusters (clusters 1, 4, 5, 8, 10); three isolates were grouped together to form cluster 5 (Figure 3). The percentages of nucleotide identity among the *msa-2b* gene ranged from 71.6% to 100%, while the percentages of amino acid identities ranged from 54.4% to 100% (Supplementary Table S2). Most sequences were grouped together with sequences from the Philippines, and formed three branches (clusters 1, 4, 5), except for one (MT113057) with a sequence from Australia, which formed cluster 10. However, three Chinese sequences formed a unique branch (cluster 5), with nucleotide identity of 100%.

**Figure 4:**
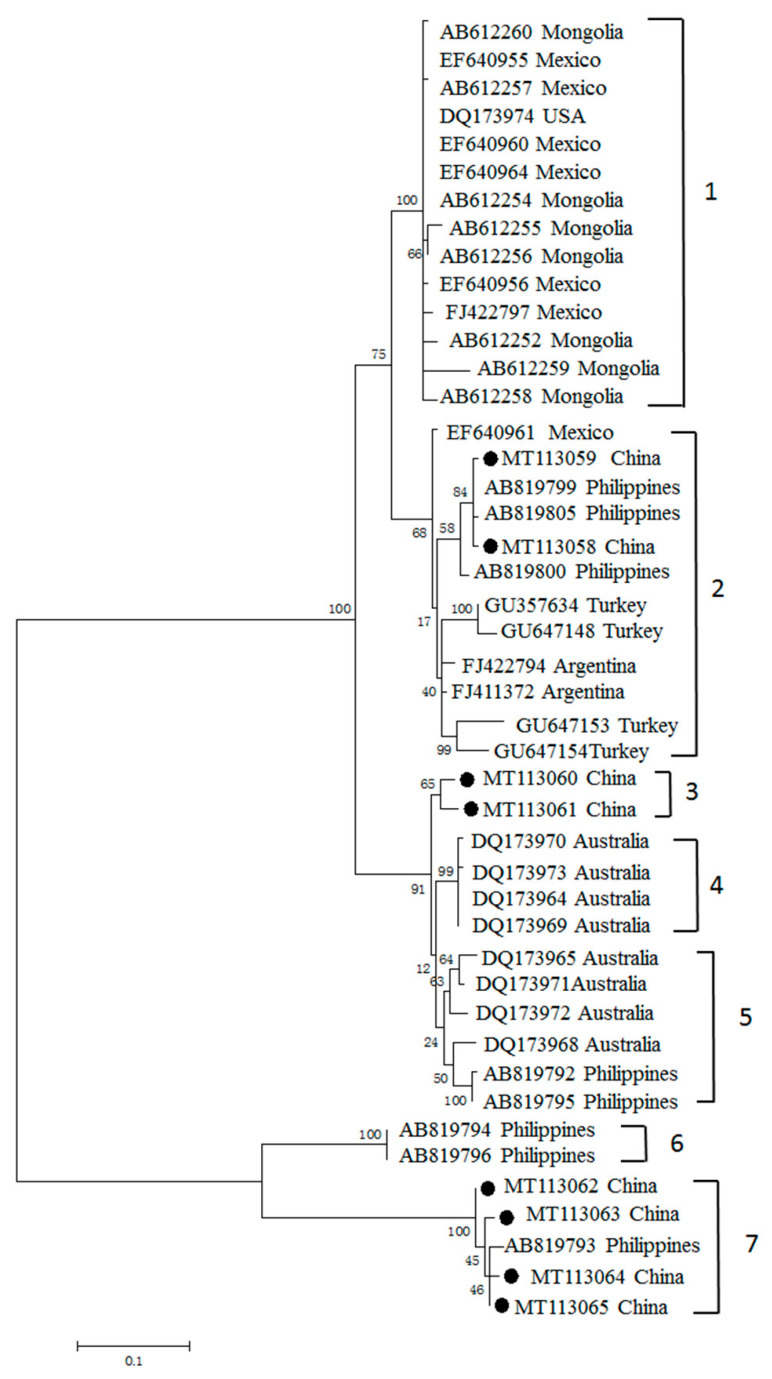
Phylogenetic tree constructed based on the predicted amino acid sequences of *B. bovis* MSA-2c deposited in the GenBank. The MSA-2c sequences obtained in this study are labeled with black circles.

To investigate the phylogenetic relationships among MSA-2c sequences, sequence alignment and cladogram analysis was performed. Our phylogenetic analysis divided MSA-2c into seven clades (Figure 4). Together with sequences previously reported from the Philippines, Turkey and Australia, the newly generated sequences were distributed across three clades (clades 2, 3 and 7). Of these three clades, clade 3 was composed exclusively of the Chinese sequences, with 98.9% sequence similarity. The percentages of nucleotide identity among the newly generated *msa-2c* ranged from 65.1% to 100%, and the percentages of amino acid identities ranged from 53.2% to 99.2% (Supplementary Table S3).

## 4. Discussion

Bovine babesiosis was first documented early in 1948 in China, and these cases were caused by two *Babesia* species, *B. bovis* and *B. bigemina* [3,6]. Subsequently, sporadic reports of this disease were made in 16 provinces across China. Based on the published literature and data obtained in this study, systematic epidemiological investigations have been performed in 16 provinces, and the available results revealed that *B. bovis* was prevalent in 14 provinces [4,5,29]. The *B. bovis* infection rate in 14 provinces where bovine babesiosis is a problem ranged from 1% to 54.9%, suggesting that the discrepancies of *B. bovis* prevalence could be due to sampling season, sampling locations, the distribution of potential tick vectors in the investigated regions, and the approaches used in the studies. Given that *B. bovis* is notorious for its high pathogenicity in susceptible cattle, and its wide distribution in China, more than 10 million cattle are at risk of bovine babesiosis caused by this pathogen.

In our present study, 575 field blood samples were collected from cattle in 10 provinces, and screened for the presence of *B. bovis* using a nPCR as previously reported [27]. Although the positive rate of *B. bovis* infection varied among the provinces investigated, *B. bovis*-specific DNA was detected in all locations. Among the 51 *B. bovis* positive samples, *msa-1*, *msa-2b* and *msa-2c* genes were successfully amplified from 23 samples. The low concentration of *B. bovis* DNA in the field samples, and the differences in the sensitivity of the nPCR and MSA PCR assays, may be reasons for the negative results of MSA amplification [27]. In addition, sequence variations in primer binding regions might be another reason for this observation.

Antigenic polymorphism has been applied as a strategy by most protozoan parasites to escape the immune response of mammalian hosts, and it is a major obstacle preventing the development of effective vaccines against these parasites [7]. In our present study, analysis of the genetic relationships of *B. bovis* MSA-1, MSA-2b and MSA-2c of Chinese isolates, with respect to almost all available related sequences deposited in the GenBank database (including Mongolia, Vietnam, Philippines, Thailand, Israel, Vietnam, Sri Lanka, Mexico, Argentina, Brazil, Japan and USA) was conducted [7,12,18,19,20,21,22,23,24,26,30,31]. Phylogenetic analysis showed that the MSA-1 amino acid sequences had a wide distribution in four clades. Clade 3 is composed of Chinese and Mexican isolate sequences, while the rest of the Chinese sequences grouped in Chinese-only separate clusters, suggesting the MSA-1 sequence is much more highly variable, and that most sequences consist of “unique antigen variants”, which are distinct from the MSA-1 sequence previously reported and are grouped in separate clades. In contrast, analysis of the genetic diversity of MSA-2b and MSA-2c revealed that MSA-2b clustered with sequences from the Philippines and Australia, while MSA-2c illustrated a close relationship with isolate sequences of Mexico, Philippines, Turkey and Argentina.

Three clades (clades 1, 7 and 8) derived from MSA-1 were exclusively composed of amino acid sequences derived from China. Chinese *B. bovis* MSA-1 exhibited three unique antigen variants, which were distinct from those found in other counties. Similar situations were also observed in the analyses of the genetic diversity of MSA-2b and MSA-2c amino acid sequences, where one and two distinct antigen variants were detected, respectively. However, we cannot conclude that the antigen variants identified in this study are exclusively found in China. Given that only a few phylogenetic studies on MSA-1, MSA-2b and MSA-2c have been performed, further worldwide epidemiological surveys and genetic diversity analyses would provide more information on our “unique antigen variants”, and the phylogenetic relationships among the field isolates.

To identify the co-infection of different *B. bovis* strains, three clones of each MSA gene amplicon were sequenced. Phylogenetic analysis revealed that sequences derived from the same sample were identical to each other, except for one, which was observed to bear variants 1 (MT113042) and 8 (MT113050). This situation is consistent with that previously reported in other protozoa, such as *Theileria parva* and *T. annulata*, and similar results for *B. bovis* were also observed in Zambia and Turkey [32,33,34]. When multiple *B. bovis* strains with distinct MSA-1 variants are transmitted by vector ticks, a new variant could probably be generated within ticks’ mid-gut via genetic exchange, which might be the reason for the emergence and expansion of new variants in cattle populations [32]. The clinical significance of infection with distinct *B. bovis* variants has not been recorded; however, it was confirmed that several variants of *T. orientalis* and *B. rossi* were closely associated with outbreaks of theileriosis and babesiosis, respectively [35,36,37]. The relationship between genetic variants in the *MSA* genes and clinical status remains unknown, and requires investigation.

In conclusion, we report that genetic diversity among *B. bovis* MSA-1, MSA-2b and MSA-2c amino acid sequences was not uncommon in endemic regions in China. Our study shows a high genetic diversity in *msa* genes among *B. bovis* populations, that could not be associated with geographic origin. Several novel genotypes of *B. bovis* were observed, and co-infection with two *MSA-1* antigen variants was detected in one infected cattle. Our data provide vital information for increasing knowledge of the genetic diversity of *B. bovis* in China, which will also be helpful for studying the global phylogenetic relationships of this crucial veterinary parasite.

## Figures and Tables

**Table 1:** Screening results of *B. bovis* positive samples.

Province	No. of Samples	No. of Positive Samples (%)
Hainan	59	6 (10.2%)
Qinghai	39	3 (7.7%)
Guangxi	71	1 (1.4%)
Guangdong	59	9 (15.3%)
Gansu	94	5 (5.3%)
Heilongjiang	33	3 (9.1%)
Chongqing	51	5 (9.8%)
Shandong	49	2 (4.1)
Henan	56	1 (1.8%)
Inner Mongolia	64	16 (25%)
Total	575	51 (8.9%)

**Table 2:** Sequence information for amplified MSA-1, MSA-2b, and MSA-2c genes.

Province	Sample ID	Gene Accession No.		
		*MSA-1*	*MSA-2b*	*MSA-2c*
Hainan	Han2	MT113038	MT113051	
	Han13		MT113052	MT113058
	Han4	MT113039		MT113059
Qinghai	QH19		MT113053	
Guangxi	GXBS3	MT113040	MT113054	
Guangdong	GDMM23	MT113041		MT113060
	GDMM44	MT113042, MT113050		
	GDMM53			MT113061
	GDMM59	MT113043		
Gansu	GSYZ17	MT113044		MT113062
	GSQL1326		MT113055	
	GSQL1330			MT113063
Heilongjiang	HLJ795	MT113045		
	HLJL22	MT113046		
Chongqing	CQBY32		MT113056	
	CQJJ11			MT113064
Shandong	SDJN7	MT113047		
	SDJN16		MT113057	
Inner Mongolia	IM27	MT113048		
	IM39	MT113049		
	IM7	MT113050		MT113065

## Supplementary Materials

The following are available online at https://www.mdpi.com/2076-0817/9/6/473/s1, Table S1: Percent Similarity of MSA-1 nucleotide and amino acid sequences, Table S2: Percent Similarity of MSA-2b nucleotide and amino acid sequences, Table S3: Percent Similarity of MSA-2c nucleotide and amino acid sequences, File S1: Amino acid sequences alignment of MSA-1, File S2: Amino acid sequences alignment of MSA-2b, File S3: amino acid sequences alignment of MSA-2.
