# Supplementary material for: Genetic Diversity of Babesia bovis MSA-1, MSA-2b and MSA-2c in China
Source: Pathogens. 2020 Jun 15;9(6):473. doi: 10.3390/pathogens9060473 (PMC7350327; doi:10.3390/pathogens9060473)
Supplement: Supplementary file 1 [file pathogens-09-00473-s001.zip › supplementary proof/Table S1.docx]

Table S1 Percent Similarity of MSA-1 nucleotide and amino acid sequences.

|  | MT113038 | MT113039 | MT113040 | MT113041 | MT113042 | MT113043 | MT113044 | MT113045 | MT113046 | MT113047 | MT113048 | MT113049 | MT113050 |
| --- | --- | --- | --- | --- | --- | --- | --- | --- | --- | --- | --- | --- | --- |
| MT113038 | 100 | 99.8 | 99.7 | 99.6 | 99.3 | 87.2 | 87.3 | 64.6 | 64.6 | 64.6 | 63.7 | 63.5 | 63.4 |
| MT113039 | 100 | 100 | 99.7 | 99.6 | 99.2 | 87.2 | 87.3 | 64.5 | 64.5 | 64.5 | 63.8 | 63.7 | 63.5 |
| MT113040 | 100 | 100 | 100 | 99.5 | 99.1 | 87.3 | 87.4 | 64.7 | 64.7 | 64.7 | 64 | 63.9 | 63.8 |
| MT113041 | 99.4 | 99.4 | 99.4 | 100 | 99 | 87 | 87.1 | 64.4 | 64.4 | 64.4 | 63.7 | 63.5 | 63.4 |
| MT113042 | 98.7 | 98.7 | 98.7 | 98.1 | 100 | 87 | 87.1 | 65 | 65 | 65 | 64 | 63.9 | 63.8 |
| MT113043 | 79.4 | 79.4 | 79.4 | 78.7 | 79 | 100 | 99.7 | 62.8 | 62.8 | 62.8 | 62.8 | 62.7 | 62.6 |
| MT113044 | 80 | 80 | 80 | 79.4 | 79.7 | 99.4 | 100 | 62.9 | 62.9 | 62.9 | 62.9 | 62.8 | 62.7 |
| MT113045 | 45.9 | 45.9 | 45.9 | 45.9 | 46.6 | 44.5 | 45.2 | 100 | 99.6 | 99.4 | 95.1 | 94.9 | 94.8 |
| MT113046 | 45.9 | 45.9 | 45.9 | 45.9 | 46.6 | 44.9 | 45.5 | 99 | 100 | 99.8 | 95.5 | 95.3 | 95.2 |
| MT113047 | 45.9 | 45.9 | 45.9 | 45.9 | 46.6 | 44.9 | 45.5 | 98.7 | 99.7 | 100 | 95.5 | 95.3 | 95.2 |
| MT113048 | 45.5 | 45.5 | 45.5 | 45.5 | 45.1 | 44.1 | 44.7 | 92.5 | 93.5 | 93.8 | 100 | 99.8 | 99.7 |
| MT113049 | 45.1 | 45.1 | 45.1 | 45.1 | 44.8 | 43.8 | 44.4 | 91.9 | 92.8 | 93.2 | 99.4 | 100 | 99.7 |
| MT113050 | 45.8 | 45.8 | 45.8 | 45.8 | 45.5 | 44.1 | 44.7 | 91.9 | 92.8 | 93.2 | 99.4 | 99.4 | 100 |

The amino acid sequence similarity values are highlighted in gray. Comparisons were conducted using the EMBOSS needle program.
