# Supplementary material for: Genetic Diversity of Babesia bovis MSA-1, MSA-2b and MSA-2c in China
Source: Pathogens. 2020 Jun 15;9(6):473. doi: 10.3390/pathogens9060473 (PMC7350327; doi:10.3390/pathogens9060473)
Supplement: Supplementary file 1 [file pathogens-09-00473-s001.zip › supplementary proof/Table S2.docx]

Table S2 Percent Similarity of MSA-2b nucleotide and amino acid sequences.

|  | MT113051 | MT113052 | MT113053 | MT113054 | MT113055 | MT113056 | MT113057 |
| --- | --- | --- | --- | --- | --- | --- | --- |
| MT113051 | 100 | 79.1 | 78.6 | 74 | 74.1 | 74.1 | 76.3 |
| MT113052 | 67.1 | 100 | 77.5 | 72 | 72.1 | 72.1 | 74.4 |
| MT113053 | 64.6 | 57.5 | 100 | 71.6 | 71.7 | 71.7 | 72.9 |
| MT113054 | 60.8 | 56 | 54.4 | 100 | 99.9 | 99.9 | 73.8 |
| MT113055 | 61.3 | 56.4 | 54.8 | 99.6 | 100 | 100 | 73.9 |
| MT113056 | 61.3 | 56.4 | 54.8 | 99.6 | 100 | 100 | 73.9 |
| MT113057 | 63.2 | 62.8 | 56.2 | 64.4 | 64.9 | 64.9 | 100 |

The amino acid sequence similarity values are highlighted in gray. Percentages identities of sequences were conducted using the EMBOSS needle program.
