# Supplementary material for: Genetic Diversity of Babesia bovis MSA-1, MSA-2b and MSA-2c in China
Source: Pathogens. 2020 Jun 15;9(6):473. doi: 10.3390/pathogens9060473 (PMC7350327; doi:10.3390/pathogens9060473)
Supplement: Supplementary file 1 [file pathogens-09-00473-s001.zip › supplementary proof/Table S3.docx]

Table S3 Percent Similarity of MSA-2c nucleotide and amino acid sequences.

|  | MT113058 | MT113059 | MT113060 | MT113061 | MT113062 | MT113063 | MT113064 | MT113065 |
| --- | --- | --- | --- | --- | --- | --- | --- | --- |
| MT113058 | 100 | 99.6 | 91.8 | 91.8 | 67.2 | 67.2 | 67.1 | 67.5 |
| MT113059 | 99.2 | 100 | 91.6 | 91.6 | 66.9 | 66.9 | 66.8 | 67.2 |
| MT113060 | 87 | 87 | 100 | 98.6 | 65.4 | 65.4 | 65.3 | 65.7 |
| MT113061 | 86.6 | 86.6 | 97.1 | 100 | 65.3 | 65.3 | 65.1 | 65.5 |
| MT113062 | 54 | 53.6 | 54.4 | 53.6 | 100 | 100 | 99 | 99.4 |
| MT113063 | 53.6 | 53.2 | 54 | 53.2 | 98.3 | 100 | 99 | 99.4 |
| MT113064 | 54 | 53.6 | 54 | 53.2 | 97.9 | 97.9 | 100 | 99.6 |
| MT113065 | 54.4 | 54 | 54.9 | 54 | 98.7 | 98.7 | 99.2 | 100 |

The amino acid sequence similarity values are highlighted in gray. Percentages identities of sequences were conducted using the EMBOSS needle program.
