# Supplementary figures and images for: Genetic Diversity of Babesia bovis MSA-1, MSA-2b and MSA-2c in China
Source: Pathogens. 2020 Jun 15;9(6):473. doi: 10.3390/pathogens9060473 (PMC7350327; doi:10.3390/pathogens9060473)

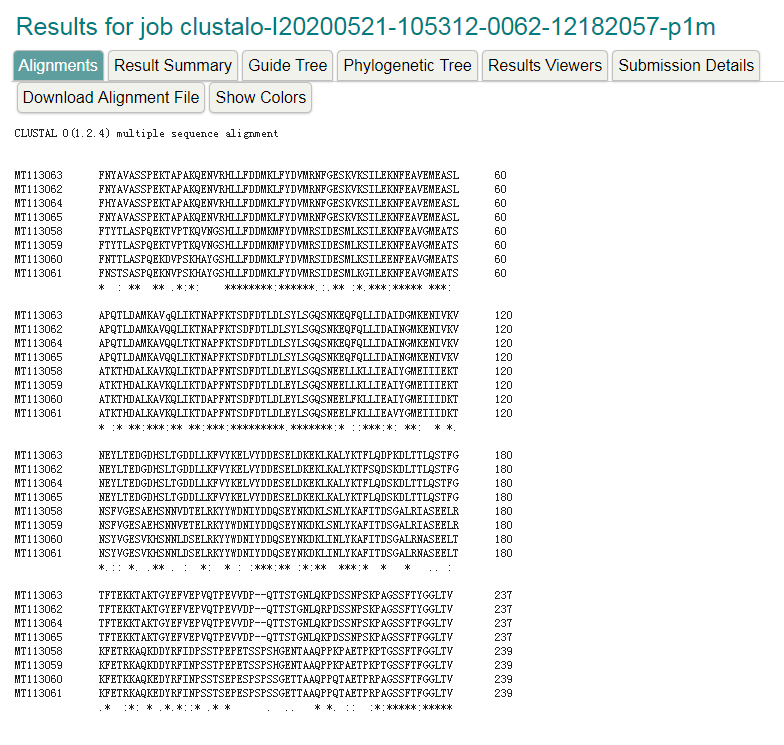


Figure 3 MSA-2c amino acid sequence alignment.

Supplement: Supplementary file 1 [file pathogens-09-00473-s001.zip › supplementary proof/File 3.docx]

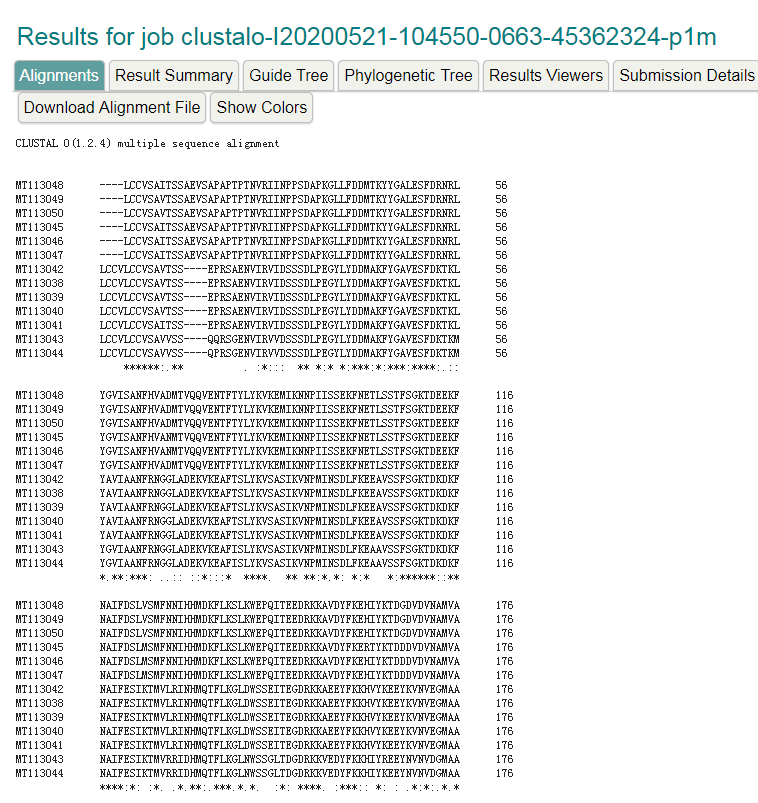


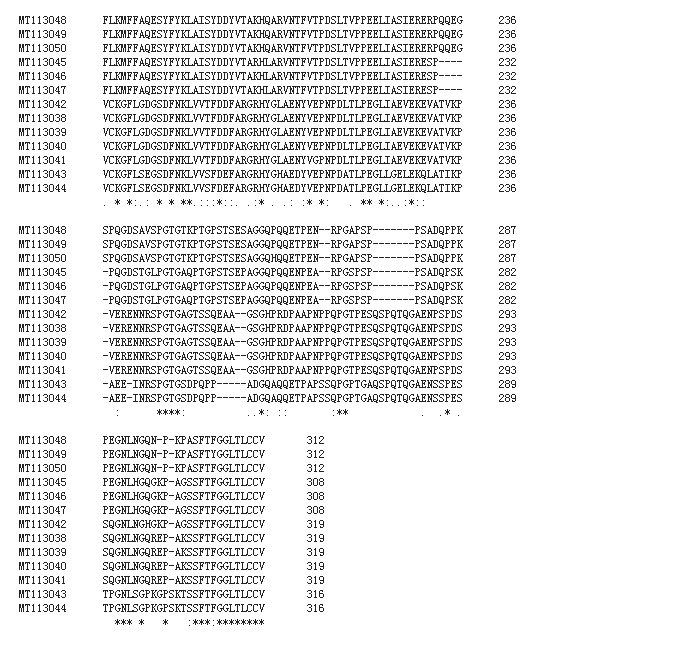


Figure 1 MSA-1 amino acid sequence alignment.

Supplement: Supplementary file 1 [file pathogens-09-00473-s001.zip › supplementary proof/File1.docx]

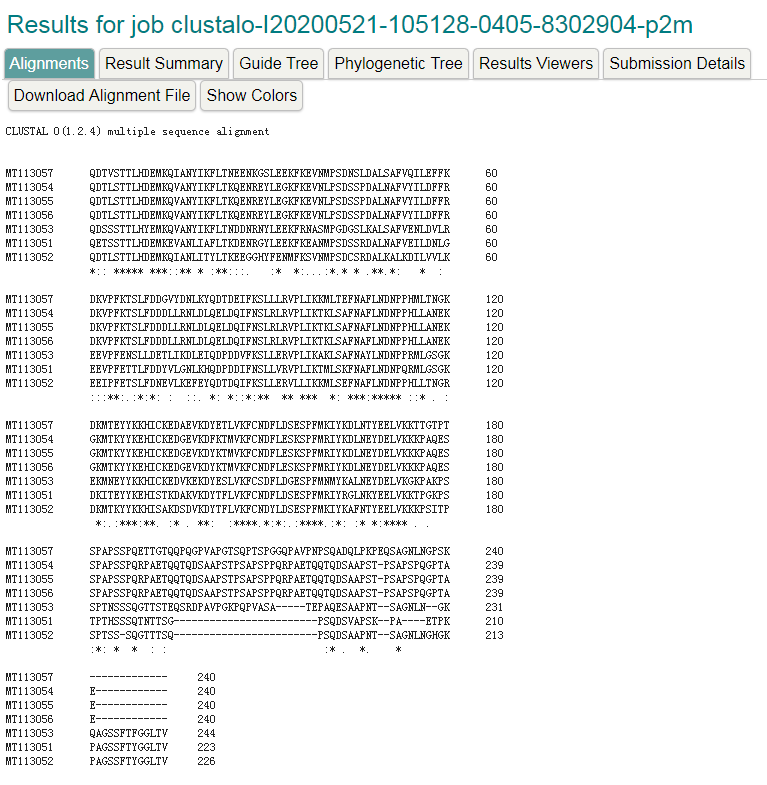


Figure 2. MSA-2b amino acid sequence alignment.

Supplement: Supplementary file 1 [file pathogens-09-00473-s001.zip › supplementary proof/File2.docx]
